# Catalytic Reduction of Organic Dyes by Multilayered Graphene Platelets and Silver Nanoparticles in Polyacrylic Acid Hydrogel

**DOI:** 10.3390/ma14092274

**Published:** 2021-04-28

**Authors:** Hanyuan Ding, Dexi Nie, Naiyuan Cui, Kaili Li, Xiaojing Zhang, Lei Zhang

**Affiliations:** 1 Ministry of Education Key Laboratory for Non-Equilibrium Synthesis and Modulation of Condensed Matter, School of Physics, Xi’an Jiaotong University, Xi’an 710049, China; haotaiyuan1111@163.com (H.D.); niedexi19970709@stu.xjtu.edu.cn (D.N.); likaaili2013@stu.xjtu.edu.cn (K.L.); jingfang114love@xjtu.edu.cn (X.Z.); 2 Beijing Institute of Spacecraft Environment Engineering, Beijing 100094, China; yuanyuanpenny@stu.xjtu.edu.cn

**Keywords:** multilayered graphene platelets, hydrogel composites, silver nanoparticles, methylene blue, catalysis

## Abstract

Graphene oxide has been widely used in the oxidative degradation of environmental pollutants. However, its catalytic role can be questioned as graphene oxide with oxygen-containing functional groups may also act as reactant in oxidative reactions. Herein, hydrogel composites loaded with multilayered graphene platelets showed excellent catalytic performance for the reduction of a wastewater organic pollutant (methylene blue) under NaBH_4_, which proved the catalytic role of multilayered graphene platelets. The liquid-based direct exfoliation method was used to prepare two-dimensional materials, which is compatible with other liquid phase methods to prepare nanomaterials. Hydrogel composites composed of multilayered graphene platelets, silver nanoparticles, and polyacrylic acid hydrogels were synthesized in water solution under irradiation with ultraviolet light, demonstrating the advantages of synthesizing nanocomposites using the liquid-based direct exfoliation method.

## 1. Introduction

Graphene is assembled by planar sp^2^ hybrid carbon atoms with large conjugated π bonds. Its unique structure endows graphene with a large specific surface area, excellent mechanical strength, high electrical and remarkable thermal conductivities, and outstanding optical and electrochemical properties [1,2,3]. Graphene-based materials, with high specific surface area, are effective for supporting various types of compounds, such as drugs, metals, bioactive, fluorescent, and electrochemically active molecules. Therefore, they are suitable for the transportation and controlled release of active principles in biomedical and tissue engineering applications and as efficient catalyst supports in environmental applications [4]. Due to its uniform and symmetrical electronic structure, pristine graphene is chemically inert. The zero-bandgap of graphene diminishes its catalytic activity and limits its use broader range of applications [2]. Therefore, significant efforts have been devoted to improving graphene performance by activation, e.g., by doping it with heteroatoms, decorating it with functional groups, applying mechanical strain, and creating vacancy defects on graphene [5]. The active catalytic edge sites and the induced oxygen-containing functional groups at the edges and defect sites are believed to make graphene-based materials serve as metal-free catalysts [6]. Metal nanoparticles have limited reserves, are expensive, and show low chemical stability. In contrast, the advantages of graphene oxide platelets are low loss, the high adsorption capacity of contaminant molecules via π–π stacking interactions, and rapid electron transfer. These advantages have been widely studied and applied in many important catalytic oxidation reactions [4,6,7]. The most common way to obtain graphene oxide (GO) is the Hummers method, because of its high oxygenated level and fast synthesis process [2,7,8,9]. However, this method usually introduces significant manganese-based impurities by adding KMnO_4_, making the consistent large-scale fabrication difficult [3]. Moreover, Presolski, and Pumera [7] showed that it is not clear if GO acts as a catalyst or reactant in catalytic oxidative reactions. Liquid-based direct exfoliation strategies to produce two-dimensional (2D) platelets from bulk layered materials in liquid media without chemical oxidation have gained significant attention owing to their extraordinary versatility, process scalability, and sustainable routes [10]. 2D platelets can be exfoliated from their bulk structures upon the collapse of bubbles or voids in solutions when ultrasonic waves are applied to the liquid [10,11,12]. If suitable solvent with matching solubility parameters are applied in liquid phase exfoliation, the obtained colloidally stable dispersions could avoid reaggregation [13]. These 2D platelets are compatible with other solution-phase-based fabrication processes because they are dispersed in suitable solvents after exfoliation and can be easily utilized to fabricate composite materials to improve their performance.

Hydrogels have properties similar to those of living matter, containing a three-dimensional (3D) polymer network in a liquid (often water molecules), and are promising materials to bridge the gap between biology and technology [14,15,16]. 3D scaffold hydrogels facilitate molecule migration and electron transfer. Additionally, the functional groups on the polymers of the hydrogel can also anchor other nanomaterials useful for catalytic applications [17]. Hydrogels have been used in tissue engineering, drug delivery, cell encapsulation, bio-separation, flexible sensors, and catalysis applications [18]. Liquid-based direct exfoliation methods can be used to bridge hydrogels with 2D materials. The combination of hydrogels with 2D materials improves performance and opens up new applications for both materials. The strategy of combining 2D multilayered graphene platelets (MLGPs) with 3D hydrogels improves their adsorption capacity and catalytic performance. Compared with catalytic 3D graphene-based materials, the MLGPs 3D hydrogel composite also has the potential to improve the reactivity of MLGPs and optimize the structure of the hydrogel by modifying the MLGPs and hydrogel, respectively. In this work, MLGPs prepared by the liquid-based direct exfoliation method were mixed with a hydrogel precursor to synthesize a MLGP/PAA hydrogel using light-induced polymerization. To illustrate the advantages of the liquid-based direct exfoliation method, which can bridge up 2D materials with nanomaterials and improve the catalytic performance of the hydrogel composite, we further prepared MLGP/Ag^0^/PAA hydrogels by adding silver nitrate to a mixture of MLGPs solution and PAA precursor. Additionally, methylene blue (MB) and 4-nitrophenol (4-NP) were selected as examples of catalytic reductive reactions because they are widely used in industry and can cause harmful health and serious environmental issues [19,20,21].

## 2. Materials and Methods

### 2.1. Materials and Reagents

Graphite (99.95%, 750–850 mesh) was purchased from Rhawn Co., Ltd. (Shanghai, China). Acrylic acid (AA ≥99%) was purchased from Fuchen Chemical Reagent Co., Ltd. (Tianjin, China). N, N′-Methylenebisacrylamide (MBAA ≥97%), α-ketoglutaric acid (≥99%), N-methyl pyrrolidone (NMP), isopropanol (IPA), and sodium borohydride (NaBH_4_, ≥99%) were purchased from Aladdin Co., Ltd. (Shanghai, China). Silver nitrate (AgNO_3_, AR) was purchased from Sinopharm Chemical Reagent Co., Ltd. (Shanghai, China). 4-nitrophenol (4-NP, ≥99%) was purchased from Macklin Chemical Reagent Co., Ltd. (Shanghai, China). Methylene Blue (MB ≥98%) was purchased from Solarbio Co., Ltd. (Beijing, China). Deionized water (18.25 MΩ⸱cm) was used as the solvent to prepare the MLGP/PAA hydrogel composite. All chemicals used in the experiments were used without further purification.

### 2.2. Preparation Process of MLGPs

**Figure 1:**
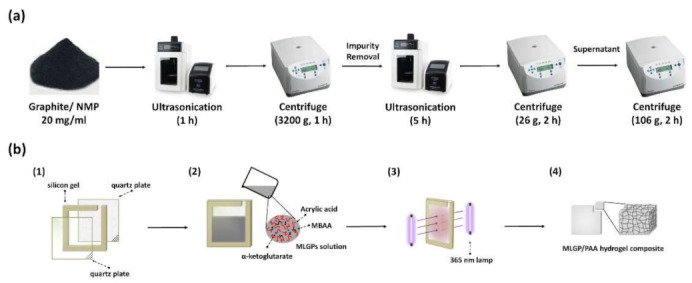
(**a**) Liquid-based direct exfoliation method to fabricate MLGPs. (**b**) Synthesis of MLGP/PAA hydrogels.

Figure 1a shows the MLGPs preparation method used in this work and was as follows. First, 300 mg of graphite were dissolved in 15 mL of NMP, and the solution was sonicated using a horn tip sonicator (JY92-2D, Scientz, Ningbo, China) at a power of 450 W for 2 h. The dispersion was then centrifuged at 3220× *g* for 1 h, and the supernatant was discarded to remove potential contaminants from the pristine graphite powder. Second, the sediment was redispersed in fresh NMP (15 mL) and sonicated under the same conditions described above for 5 h to produce MLGPs. Third, to obtain relatively uniform MLGPs of different sizes and thicknesses, liquid cascade centrifugation was applied to separate the larger and smaller MLGPs. The above solution was centrifuged at 26× *g* for 2 h to remove the largest platelets and aggregates. The supernatant was further centrifuged at 106× *g* for 2 h to remove small platelets. The sediment was then dispersed in 15 mL of IPA solution for further use (normal boiling point of 82 °C).

### 2.3. Preparation Process of MLGP/PAA Hydrogel and MLGP/Ag^0^/PAA Hydrogel

In a typical procedure (Figure 1b), AA (4.32 g), MBAA (0.1848 g), and α-ketoglutaric acid (0.003 g) were added to 10 mL of deionized water at room temperature (~25 °C). The solution was sonicated for 3 min to uniformly disperse the additives in an ultrasonic water bath. Then, 2 mL of the MLGPs dispersion was mixed into the above solution and sonicated for 2 min. The precursor solution was then dropped into a container consisting of two quartz glasses and a 3 mm silicone pad. Two UV lamps (TL-K 40 W ACTINIC BL, Philips, Hamburg, Germany) with a peak wavelength of 365 nm were used to induce polymerization. MLGP/PAA hydrogels were prepared after 10 h of irradiation. The preparation of MLGP/Ag^0^/PAA hydrogels was accomplished using the procedure described above, in which an additional 0.17 g AgNO_3_ were added to the hydrogel monomers in water at the beginning. The distance between the lamp and precursor solution was ~7 cm, which we found is the best distance for producing the silver nanoparticles with desired size (i.e., ~1.3 nm).

### 2.4. Characterization Techniques

Transmission electron microscopy (TEM) and high-resolution transmission electron microscopy (HRTEM) were carried out on a JEM-2100 (JEOL, Akishima, Tokyo, Japan) instrument operating at an accelerating voltage of 200 kV. Scanning electron microscopy (SEM) images were obtained using EVO-10 microscope (Zeiss, Munich, Germany). Atomic force microscopy (AFM) was conducted using SPM-9700HT (Shimadzu, Kyoto, Japan). Raman spectra were obtained on a DXR2xi Raman spectrometer (Thermo Fisher, Waltham, MA, USA) with 514.5 nm wavelength incident laser light. X-ray photoelectron spectroscopy (XPS) studies were performed on a ESCALAB Xi^+^ spectrometer (Thermo Fisher, Waltham, MA, USA) using an Al Kα X-ray source. Infrared spectra (IR) were recorded on a micro-infrared spectrometer (Vertex70, Bruker, Germany). X-ray Diffraction (XRD) spectra were obtained on a D8 Advance spectrometer (Bruker, Bremen, Germany).Thermogravimetric analysis (TGA) was performed at a heating rate of 10 K/min under a nitrogen atmosphere (TGA/DSC 3+, Mettler Toledo, Columbus, OH, USA). UV-VIS absorption spectra were recorded on a UV-VIS/NIR spectrophotometer (Lambda-950, PerkinElmer, Waltham, MA, USA).

### 2.5. Catalytic Experiment

The catalytic properties of the as-prepared MLGP/PAA and MLGP/*Ag*^0^/PAA hydrogels were evaluated as follows. To 2 mL of an aqueous solution of MB or 4-NP (0.1 mM), 0.4 mL of freshly prepared NaBH_4_ solution (0.1 mM) was added, in a 50 mL beaker, and kept for 15 min. Then, a piece of hydrogel composite (1 cm × 1.5 cm) was added to the above solution to initiate the catalytic reaction. The hydrogel was removed from the reaction solution at a certain time interval, and the absorption spectrum of the solution was measured using the UV-VIS/NIR spectrophotometer.

## 3. Results and Discussion

### 3.1. Preparation of MLGPs by Liquid-Based Direct Exfoliation

The tip sonication method was applied instead of a normal ultrasonication bath to enhance the exfoliation of graphite. High-intensity ultrasonic waves cause the cavitation of small vacuum bubbles or voids in the liquid, resulting in very high local shear strains [10]. Once the shear strain force exceeds the van der Waals interaction between the graphite layers, graphite can be exfoliated into a few layers or a single layer. Normally, the position of the tip, the power of ultrasonic waves, and processing time determine the quality of the resultant products. The size of the prepared platelets is small if the power is too high to break the C–C covalent bonds and it is also affected by the type of liquid solvent [22]. Efficient exfoliation is achieved if the surface energy of the solvent matches that of the solute. NMP was chosen as a solvent to exfoliate graphite because the surface energy of NMP is 70 mJ cm^−2^ [10], which agrees with the surface energy of graphene (~70 mJ cm^−2^). The detailed experimental procedure was described above. Since tip sonication induces heterogeneous forces in the solution, obtaining platelets of uniform size and thickness is not possible. Therefore, a centrifugation step is necessary to filter relatively uniform platelets after sonication. In our experiments, MLGPs were prepared during the sonication exfoliation process and the observed oxygen-containing functionalities were derived from air [23].

### 3.2. Preparation of MLGP/PAA Hydrogels and MLGP/Ag^0^/PAA Hydrogels

As IPA is miscible with water, the precursor solution of the PAA hydrogel became as visible as a uniform light-gray solution after the addition of the MLGPs solution. Ultraviolet light was applied to provide free radicals inducing the polymerization of acrylic acid monomers and forming the PAA hydrogels [24], while the well-dispersed MLGPs were uniformly distributed in the 3D scaffolds of PAA.

To demonstrate the advantages of the liquid-based direct exfoliation method to fabricate nanocomposites and enhance the catalytic performance of the hydrogel, we further applied silver nitrate to fabricate MLGP/*Ag^0^*/PAA hydrogels. The detailed fabrication process was described above. The procedure is based on a one-pot synthesis process, benefiting from the radicals provided by ultraviolet light to polymerize the hydrogel as well as to reduce silver nanoparticles (*Ag^0^*) simultaneously. The reductive reaction equations of *Ag*^0^ are described by Equations (1) and (2) [25]. Additionally, silver ions can be well dispersed in the hydrogel because of the adsorption of negatively charged carboxylic groups on AA monomers.

(1) Ag++H2O→Ag0+H++O•H

(2) $$nAg^{0}\to {\left(Ag^{0}\right)}_{n}$$

### 3.3. Characterization of MLGPs Prepared from Liquid-Based Direct Exfoliation

**Figure 2:**
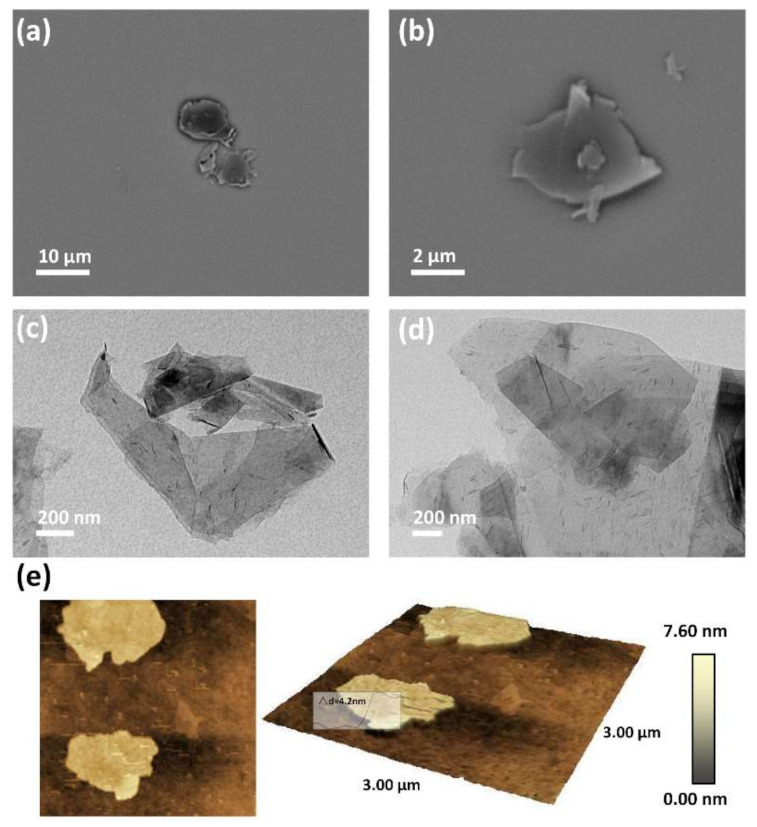
Morphology characterization of MLGPs. (**a**,**b**) SEM images at different scales; (**c**,**d**) TEM images at different scales; (**e**) AFM images.

The size of the prepared MLGPs were investigated via SEM and TEM analyses. The MLGPs solution was directly dropped onto a silicon chip and copper grind for SEM and TEM observation, respectively. Figure 2a,b shows the SEM images of the MLGPs. SEM results were consistent with the TEM images (Figure 2c,d), in which the dark areas indicate the restacking of platelets during solvent volatilization. The AFM images revealed MLGPs with a height of ~4 nm, indicating the formation of multilayered graphene platelets (Figure 2e).

**Figure 3:**
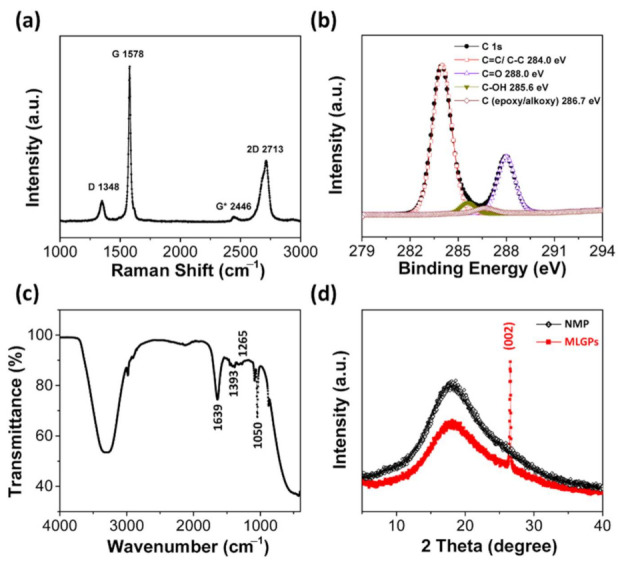
Raman (**a**), XPS (**b**), IR (**c**), and XRD (**d**) spectra of MLGPs.

Raman spectroscopy was used to investigate the structural and electronic properties of MLGPs. Four peaks were clearly observed in the Raman spectra (Figure 3a). The G band at ~1578 cm^−1^ is due to the high-frequency E_2g_ phonon at the Brillouin zone center Γ [26]. The D band at ~1348 cm^−1^, originating from transverse optic (TO) phonons around the Brillouin zone corner Κ, indicates the existence of defects [26], which were caused by the high-intensity ultrasonic waves. The 2D band in the range of 2500–2800 cm^−1^ comes from a process, in which two phonons with opposite wave vectors meet momentum conservation [26]. In Figure 3a, the intensity ratio of I_D_/I_G_ (≈0.13) and the full width at half maximum (FWHM) of G peak revealed that the disorder of the obtained platelets was in stage one [27]. The band at ~2446 cm^−^^1^ (D + D’’) is due to a D phonon and a phonon belonging to the longitudinal acoustic (LA) branch [26].

XPS was employed to characterize the oxygenated functional groups in the MLGPs. As showed in Figure 3b, the C1s deconvolution spectrum of MLGPs exhibited the characteristic peaks of the C=C/C–C skeleton, hydroxyl, epoxyl, and carbonyl groups centered at 284.0, 285.6, 286.7 and 288.0 cm^−1^, respectively [28]. As showed in Figure 3c, the measured IR spectrum of MLGPs further confirmed the existence of oxygenated functional groups (hydroxyl, epoxyl, and carbonyl groups at 1393, 1265, 1050 cm^−1^, respectively) [28,29,30]. These results confirmed that multilayered MLGPs were successfully prepared using the liquid-based direct exfoliation method. Moreover, this method can be combined with other liquid-based fabrication methods to improve catalytic performance, such as the fabrication of MLGP/*Ag*^0^/PAA hydrogels. Figure 3d shows XRD spectra of MLGPs and NMP, where a (002) peak of graphite can be observed.

### 3.4. Characterization of MLGP/PAA and MLGP/Ag^0^/PAA Hydrogels

**Figure 4:**
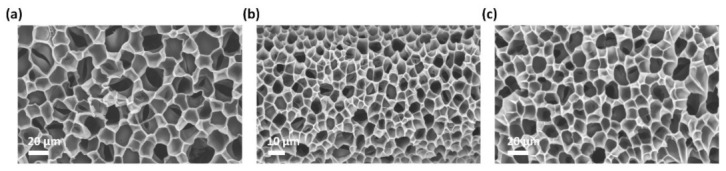
SEM images of pure PAA (**a**), MLGP/PAA (**b**), and MLGP/*Ag*^0^/PAA (**c**) hydrogels.

**Figure 5:**
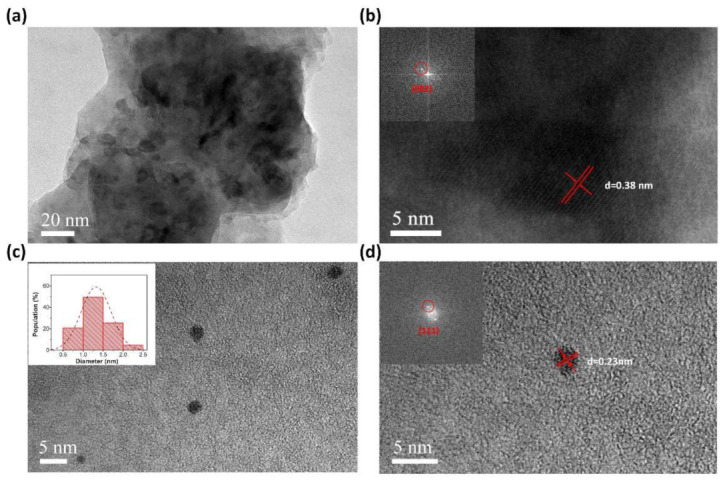
TEM images of MLGPs in MLGP/PAA hydrogel (**a**,**b**) and *Ag*^0^ in MLGP/*Ag*^0^/PAA hydrogel (**c**,**d**). The inset in (**c**) is the size histogram of 150 silver nanoparticles in MLGP/*Ag*^0^/PAA hydrogel.

The 3D scaffolds of the PAA hydrogel supported MLGPs to disperse active sites and improved mass transfer efficiency during the catalytic reactions. To demonstrate the superior compatibility of the liquid-based direct exfoliation method, we further added silver nitrate to a solution of MLGPs and PAA hydrogel precursor to fabricate MLGP/*Ag*^0^/PAA hydrogels. Figure 4 shows the SEM images of the PAA, MLGP/PAA, and MLGP/*Ag*^0^/PAA hydrogels. The addition of MLGPs and *Ag*^0^ did not disturb the final structure of the PAA hydrogel, which was only influenced by the fabrication process. The low-magnification TEM image of the as-prepared hydrogel composites in Figure 5a shows that the MLGPs were well dispersed in the PAA hydrogel scaffolds. Figure 5b shows the HRTEM image of the MLGP/PAA hydrogel. The interlayer space (d-spacing) was 0.38 nm, which is larger than the d-spacing of pristine graphite (0.34 nm (002)) due to the introduction of oxygenated functional groups. Figure 5c,d presents the HRTEM images of the silver nanoparticles in the MLGP/*Ag*^0^/PAA hydrogel. The inset of Figure 5c shows the size histogram of *Ag*^0^ in the hydrogel composite. The average size of isolated *Ag*^0^ was ~1.3 nm. The crystalline interplanar spacing of ultrafine nanoparticles was ~0.23 nm, which corresponds to the (111) crystal plane of *Ag*.

**Figure 6:**
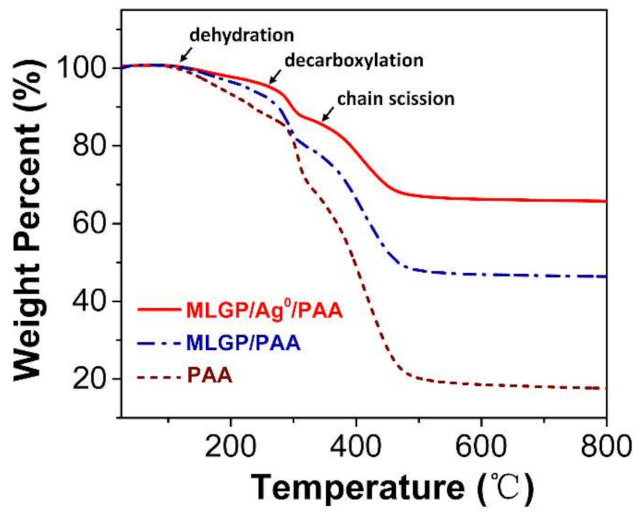
TGA curves of three different hydrogels. The brown, blue, and red lines indicate pure PAA, MLGP/PAA, and MLGP/*Ag*^0^/PAA hydrogels, respectively.

TGA was used to characterize the thermal stability and loading (wt.%) of the components in the hydrogel composites. MLGPs lose adsorbed water molecules and labile oxygen-containing functional groups at temperatures <200 °C [28]. Additionally, there are three main processes during PAA decomposition when increasing the temperature. As shown in Figure 6, the dehydration of PAA occurs when heating up to 150 °C. When the temperature is >200 °C, the intra or intermolecular reaction of carboxyl groups in PAA hydrogels occurs, leading to further dehydration. Decarboxylation becomes increasingly important at temperatures >250 °C, and chain scission reactions occur when the temperature is >350 °C [31,32,33].

**Figure 7:**
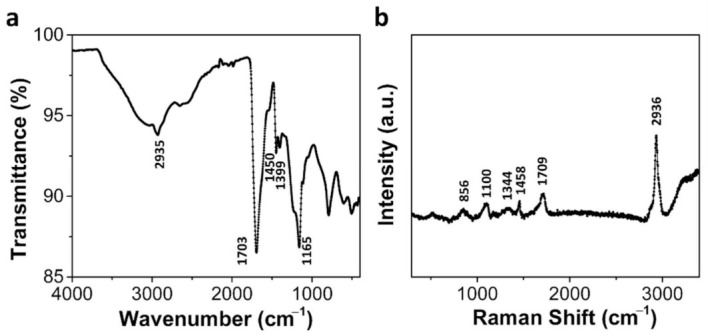
IR (**a**) and Raman spectrum (**b**) of the MLGP/PAA hydrogel.

Figure 7 shows the IR and Raman spectra of the MLGP/PAA hydrogel. The IR spectrum of the MLGP/PAA hydrogel was similar to that of the pure PAA hydrogel, which indicated that the MLGPs were well enclosed in the hydrogel scaffolds. Figure 7a shows a strong absorption band at 1703 cm^−1^ due to C=O stretching. It also exhibits the presence of CH_2_ or CH stretching at 2935 cm^−1^, CH_2_ deformation at 1450 cm^−1^, and C–O stretching coupled with O–H in-plane bending at 1399 and 1165 cm^−1^ [34]. The Raman spectrum of the MLGP/PAA hydrogel (Figure 7b) was also in good agreement with that of the pure PAA hydrogel. The sharp peaks at 2936, 1709, 1458, 1344, and 856 cm^−1^ indicate CH_2_ or CH stretching, C=O stretching mode, CH_2_ deformation, CH_2_ twist, and C–COOH stretching, respectively [31].

### 3.5. Catalytic Performance of MLGP/PAA and MLGP/Ag^0^/PAA Hydrogels

Graphene-based catalysts have abilities to promote the production of the sulfate radicals (SO_4_^•^–) [6]. These radicals have high oxidative potential and are used for the oxidative degradation of pollutants, such as phenols and pharmaceuticals. However, catalytic role of graphene oxide is unclear in some oxidative reactions [7]. This work verified that MLGPs in MLGP/PAA hydrogels are effective catalysts for the reduction of MB and 4-NP under NaBH_4_. Additionally, contrast experiments were carried out to distinguish the catalytic and adsorption performance of the hydrogel composites.

In MLGP/PAA hydrogels, the as-prepared MLGPs had abundant edges, defects, and functional groups, which improved catalytic performance. The basal plane of the MLGPs was also a good support for the adsorption of reactants and uniform catalyst loading. The hydrogel structure and π–π stacking interactions on MLGP caused high adsorption capacity of contaminant molecules and a high preconcentration proximal to the catalytic components. Additionally, the 3D hydrogel scaffolds supplied the active MLGPs with high dispersity, preventing the aggregation of platelets and reduced catalytic performance. The porous 3D hydrogel also facilitated the mass transfer between the hydrogel and the pollutant, which improved the catalytic efficiency.

**Figure 8:**
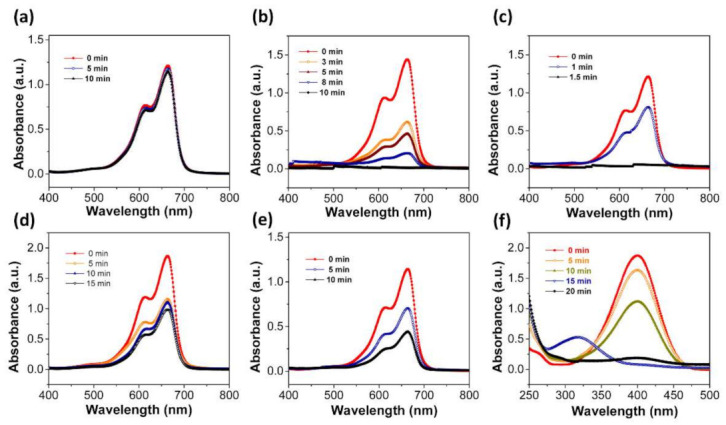
Characterization of catalytic performance of MLGP/PAA and MLGP/*Ag*^0^/PAA hydrogels. (**a**) UV-VIS spectrum of mixture solution of MB and NaBH_4_, (**b**) catalytic performance of MLGP/PAA hydrogel to reduce MB, (**c**) catalytic performance of MLGP/*Ag*^0^/PAA hydrogel to reduce MB, (**d**) pure PAA hydrogel adsorption capacity of MB, (**e**) MLGP/PAA hydrogel adsorption capacity of MB, and (**f**) catalytic performance of MLGP/PAA hydrogel to reduce 4-NP.

As showed in Figure 8a, NaBH_4_ did not react with MB without the catalyst. When MLGP/PAA hydrogel was added to the mixture of MB and NaBH_4_, the characteristic peak of MB rapidly decreased within 10 min (Figure 8b). Figure 8c shows the catalytic performance of the MLGP/*Ag*^0^/PAA hydrogel. The maximum absorption peak of MB completely disappeared within 1.5 min. Therefore, the MLGPs show catalyst and cocatalyst activity because of their excellent ability to accept and transport electrons.

Pure PAA hydrogels were placed in a mixture of MB and NaBH_4_, and the MB content remained constant after 15 min (Figure 8d). To verify the adsorption role of MLGP/PAA hydrogels, we placed a piece of MLGP/PAA hydrogel into the MB solution without NaBH_4_. MB could be adsorbed into the hydrogel composite; however, the MB content remained practically constant after 10 min, indicating adsorption saturation (Figure 8e). Therefore, the adsorption performance of MLGP/PAA hydrogel cannot completely remove MB.

The MLGP/PAA hydrogel could also catalyze the reduction of other organic dyes, such as 4-NP. When freshly prepared NaBH_4_ solution was added to the 4-NP solution, the color of the solution changed from light- to dark-yellow due to the formation of 4-nitrophenolate ions under alkaline conditions [35]. The maximum absorbance of 4-NP was shifted to 400 nm. After a piece of MLGP/PAA hydrogel was added to the 4-NP solution, BH_4_^–^ was first adsorbed on the surface of the catalyst, which increased the Fermi potential and decreased the reduction potential of the catalysts. After the transfer of electrons from BH_4_^–^ to the MLGPs, hydrogen atoms from BH_4_^–^ attack the 4-NP molecules reducing them [36,37]. Since the oxidative MLGPs also reacted with NaBH_4_, the peak shifted back to 280 nm when NaBH_4_ was consumed. When we added NaBH_4_, the reduction reaction continued, and the absorption peak at 400 nm corresponding to 4-NP disappeared in 20 min (Figure 8f).

## 4. Conclusions

In this work, a liquid-based direct exfoliation method was used to prepare MLGPs from graphite powders. The method described in this work is a relatively low-cost and scalable process. Additionally, it is appropriate to produce MLGPs without using any acid or oxidant substances as opposed to the Hummers method. XPS, Raman, and IR spectra were used to characterize the platelets. PAA hydrogels were utilized as 3D support scaffolds for MLGPs, which also facilitated mass transfer and improved the catalytic performance. This work clarified the catalytic role of that MLGPs using MLGP/PAA and MLGP/*Ag*^0^/PAA hydrogels for the effective reduction of MB and 4-NP under NaBH_4_. Excellent catalytic performance was observed because of the abundant edges, defects, and functional groups found on the MLGPs in MLGP/PAA hydrogel. Additionally, the advantages of the liquid-based direct exfoliation method were demonstrated by preparing solid MLGP/*Ag*^0^/PAA hydrogels catalysts based on a one-pot process. Characterization results showed that the addition of MLGPs and other additives such as silver nanoparticles did not change the 3D structure of the PAA hydrogel using the liquid-based direct exfoliation method.

## Data Availability

The data presented in this study are available on request from the corresponding author.

## Abbreviations

MLGPs	Multilayered Graphene Platelets
PAA	polyacrylic acid
MLGP/PAA hydrogel	multilayered graphene platelets/polyacrylic acid hydrogel composite
*Ag* ^0^	silver nanoparticles
MLGP/*Ag*^0^/PAA hydrogel	multilayered graphene platelets/silver nanoparticles/polyacrylic acid hydrogel composite
NMP	N-methyl pyrrolidone
IPA	isopropanol
MB	methylene blue
4-NP	4-nitrophenol

## References

[1] Yang F., Deng D.H., Pan X.L., Fu Q., Bao X.H. (2015). Understanding nano effects in catalysis. Natl. Sci. Rev..

[2] Kong X.K., Chen C.L., Chen Q.W. (2014). Doped graphene for metal-free catalysis. Chem. Soc. Rev..

[3] Wang L., Pumera M. (2016). Electrochemical catalysis at low dimensional carbons: Graphene, carbon nanotubes and beyond—A review. Appl. Mater. Today.

[4] Qiu B.C., Xing M.Y., Zhang J.L. (2018). Recent advances in three-dimensional graphene based materials for catalysis applications. Chem. Soc. Rev..

[5] Zhang C., Guo N., Yam K.M. (2019). Graphene based solid-state catalysis: From computational understanding to applications. J. Mater. Sci. Nanotechnol..

[6] Komeily-Nia Z., Qu L.-T., Li J.-L. (2021). Progress in the Understanding and Applications of the Intrinsic Reactivity of Graphene-Based Materials. Small Sci..

[7] Presolski S., Pumera M. (2018). Graphene Oxide: Carbocatalyst or Reagent?. Angew. Chem. Int. Ed..

[8] Ahn Y., Oh H., Yoon Y., Park W.K., Yang W.S., Kang J.W. (2017). Effect of graphene oxidation degree on the catalytic activity of graphene for ozone catalysis. J. Environ. Chem. Eng..

[9] Zhu S.H., Wang J.G., Fan W.B. (2015). Graphene-based catalysis for biomass conversion. Catal. Sci. Technol..

[10] Niu L., Coleman J.N., Zhang H., Shin H., Chhowalla M., Zheng Z. (2016). Production of Two-Dimensional Nanomaterials via Liquid-Based Direct Exfoliation. Small.

[11] Nicolosi V., Chhowalla M., Kanatzidis M.G., Strano M.S., Coleman J.N. (2013). Liquid exfoliation of layered materials. Science.

[12] Cui X., Zhang C., Hao R., Hou Y. (2011). Liquid-phase exfoliation, functionalization and applications of graphene. Nanoscale.

[13] Backes C., Abdelkader A.M., Alonso C., Andrieux-Ledier A., Arenal R., Azpeitia J., Balakrishnan N., Banszerus L., Barjon J., Bartali R. (2020). Production and processing of graphene and related materials. 2D Mater..

[14] Yang N., Qi P., Ren J., Yu H., Liu S., Li J., Chen W., Kaplan D.L., Ling S. (2019). Polyvinyl Alcohol/Silk Fibroin/Borax Hydrogel Ionotronics: A Highly Stretchable, Self-Healable, and Biocompatible Sensing Platform. ACS Appl. Mater. Interfaces.

[15] Simon D.T., Gabrielsson E.O., Tybrandt K., Berggren M. (2016). Organic bioelectronics: Bridging the signaling gap between biology and technology. Chem. Rev..

[16] Yuk H., Lu B., Zhao X. (2019). Hydrogel bioelectronics. Chem. Soc. Rev..

[17] Rivnay J., Owens R.M., Malliaras G.G. (2014). The Rise of Organic Bioelectronics. Chem. Mater..

[18] De France K.J., Xu F., Hoare T. (2018). Structured Macroporous Hydrogels: Progress, Challenges, and Opportunities. Adv. Healthc. Mater..

[19] Mei X., Liu J., Guo Z., Li P., Bi S., Wang Y., Yang Y., Shen W., Wang Y., Xiao Y. (2019). Simultaneous p-nitrophenol and nitrogen removal in PNP wastewater treatment: Comparison of two integrated membrane-aerated bioreactor systems. J. Hazard. Mater..

[20] Rashed M.N., El Taher M.A.E.D., Fadlalla S.M.M. (2016). Adsorption of methylene blue using modified adsorbents from drinking water treatment sludge. Water Sci. Technol..

[21] Dod R., Banerjee G., Saini D.R. (2015). Removal of methylene blue (MB) dye from water environment by processed Jowar Stalk [*Sorghum bicolor* (L.) Moench] adsorbent. Clean Technol. Environ. Policy.

[22] Huo C., Yan Z., Song X., Zeng H. (2015). 2D materials via liquid exfoliation: A review on fabrication and applications. Sci. Bull..

[23] Skaltsas T., Ke X., Bittencourt C., Tagmatarchis N. (2013). Ultrasonication Induces Oxygenated Species and Defects onto Exfoliated Graphene. J. Phys. Chem. C.

[24] Elliott J.E., Macdonald M., Nie J., Bowman C.N. (2004). Structure and swelling of poly(acrylic acid) hydrogels: Effect of pH, ionic strength, and dilution on the crosslinked polymer structure. Polymer.

[25] Yonezawa Y., Sato T., Kuroda S. (1991). Photochemical Formation of Colloidal Silver: Peptizing Action of Acetone Ketyl Radical. J. Chem. Soc. Faraday Trans..

[26] Ferrari A.C., Basko D.M. (2013). Raman spectroscopy as a versatile tool for studying the properties of graphene. Nat. Nanotechnol..

[27] Cançado L.G., Jorio A., Ferreira E.H.M., Stavale F., Achete C.A., Capaz R.B., Moutinho M.V.O., Lombardo A., Kulmala T.S., Ferrari A.C. (2011). Quantifying Defects in Graphene via Raman Spectroscopy at Different Excitation Energies. Nano Lett..

[28] Zhu C., Guo S., Fang Y., Dong S. (2010). Reducing Sugar: New Functional Molecules for the Green Synthesis of Graphene Nanosheets. ACS Nano.

[29] Rattana T., Chaiyakun S., Witit-anun N., Nuntawong N., Chindaudom P., Oaew S., Kedkeaw C., Limsuwan P. (2012). Preparation and characterization of graphene oxide nanosheets. Procedia Eng..

[30] Krishnamoorthy K., Kim G.-S., Kim S.J. (2013). Graphene nanosheets: Ultrasound assisted synthesis and characterization. Ultrason. Sonochem..

[31] Dong J., Ozaki Y., Nakashima K. (1997). FTIR Studies of Conformational Energies of Poly(acrylic Acid) in Cast Films. J. Polym. Sci. Part B: Polym. Phys..

[32] De la Fuente J.L., Wilhelm M., Spiess H.W., Madruga E.L., Fernández-Garcia M., Cerrada M.L. (2005). Thermal, morphological and rheological characterization of poly(acrylic acid-g-styrene) amphiphilic graft copolymers. Polymer.

[33] Krivorotova T., Jonikaite-Svegzdiene J., Radzevicius P., Makuska R. (2014). Synthesis by RAFT polymerization and properties of anionic cylindrical molecular brushes bearing poly(acrylic acid) side chains. React. Funct. Polym..

[34] Abdollahi R., Taghizadeh M.T., Savani S. (2018). Thermal and mechanical properties of graphene oxide nanocomposite hydrogel based on poly(acrylic acid) grafted onto amylose. Polym. Degrad. Stab..

[35] Chang Y.-C., Chen D.-H. (2009). Catalytic reduction of 4-nitrophenol by magnetically recoverable Au nanocatalyst. J. Hazard. Mater..

[36] Pradhan N., Pal A., Pal T. (2002). Silver nanoparticle catalyzed reduction of aromatic nitro compounds. Colloids Surf. A.

[37] Naik B., Hazra S., Prasad V.S., Ghosh N.N. (2011). Synthesis of Ag nanoparticles within the pores of SBA15: An efficient catalyst for reduction of 4-nitrophenol. Catal. Commun..

